# Crop wild relative populations of *Beta vulgaris* as source for genome-wide association mapping of complex traits

**DOI:** 10.1007/s00122-025-04947-3

**Published:** 2025-06-21

**Authors:** Lisa Bertram, Friedrich Kopisch-Obuch, Matthias Frisch

**Affiliations:** 1 Institute of Agronomy and Plant Breeding II, Justus Liebig University, Giessen, Germany; 2 KWS SAAT SE & Co. KGaA, Einbeck, Germany

## Abstract

**Key message:**

Wild beet populations can be used for the detection of minor genes underlying quantitative traits

**Abstract:**

Wild beet populations as valuable source for new genetic variation have so far not been used to detect minor genes underlying quantitative traits, such as drought tolerance or yield. These traits cannot be assessed in wild beets per se but require the development of a beet for phenotypic evaluation. Hence, crossing to elite genome is necessary. Our objective was to determine how QTL detection is affected by (1) the properties of the wild beet population, (2) the quantitative trait architecture, and (3) the structure of the mapping populations. Based on genotypic data of three wild beet populations, nine crossing designs to construct mapping populations were simulated and evaluated for their power to detect minor QTL and their false detection rate. Mapping populations containing 50% wild beet genome have the highest power in this study and can detect even QTL with allele frequencies of < 1% with reasonable power. However, to allow for reasonable phenotyping within field trials at least 75% elite genome in the mapping population is needed. We conclude that crossing designs based on elite x wild beet F1s are most suitable for genome-wide association mapping of complex traits in wild beet populations.

## Introduction

Crop wild relatives harbor tremendous genetic diversity and carry many alleles for traits of agronomical importance, that were lost during domestication but allow them to adapt to diverse and rapidly changing environments. Hence, they are a valuable source of allelic variation for breeding programs (Fénart et al. 2008; Dempewolf et al. 2017). There are many successful examples of the use of crop wild relatives in breeding for disease and pest improvement, such as in wheat (*Triticum aestivum*), rice (*Oryza sativa*), chickpea (*Cicer arietinum*), potato (*Solanum tuberosum*), tomato (*Lycopersicon esculentum*), cassava (*Manihot esculenta*), sunflower (*Helianthus annuus*), banana (*Musa acuminate*) or lettuce (*Lactuca sativa*, Hajjar & Hodgkin 2007, Kashyap et al. 2022).

Quantitative traits are generally more difficult to detect due to their more complex inheritance. Nevertheless, there have been examples also for the use of crop wild relatives for the improvement of such traits. In example for improving tolerance to abiotic stress such as drought and cold or salt tolerance through wild relatives in rice, tomato, barley (*Hordeum vulgare*), cowpea (*Vigna unguiculata*) or chickpea (Hajjar & Hodgkin 2007; Kashyap et al. 2022). Most crop wild relatives have a poor per se agronomic performance. Therefore, they are rarely sought for when targeting yield-related traits (Hajjar & Hodgkin 2007). Nevertheless, crop wild relatives may show favorable variation even at loci for yield components (Liu et al. 2016; Xu et al. 2022). The expression of these valuable alleles, however, may be masked and the performance of crop wild relatives per se might not appear beneficial in standard breeding trials (Dempewolf et al. 2017; Bohra et al. 2022).

Several approaches were successful in overcoming the challenges presented by wild relatives by crossing to elite germplasm (pre-breeding) to increase overall performance and to enable testing of material based on wild relatives also for yield-related traits (Bohra et al. 2022; Schulthess et al. 2022). Working with such segregating populations allows for the demasking of useful genomic regions by mapping. For example, Mace et al. (2020) have described a combination of nested association mapping and backcrossing of crop wild relatives with common elite parents in sorghum (*Sorghum bicolor*). Wild x elite F1s were used to estimate the contribution of wild relatives to yield in wheat (Schulthess et al. 2022). In wheat and banana breeding cryptic variation has been found to result in significant and often unexpected superior performance of the crosses between wild relatives and the domesticated crop (Dempewolf et al. 2017). For common bean (*Phaseolus vulgaris*) wild alleles were detected, that increased the yield over the domesticated parent in backcrossed-inbred-line populations (Berny Mier y Tera et al. 2020). Combs and Bernardo (2013) used genomic selection to increase yield in exotic x elite populations of maize (*Zea mays*). Crop wild relative-derived lines of durum wheat (*Triticum durum*), barley and lentil (*Lens culinaris*) were found to outperform the used elite checks (El Haddad et al. 2021). Yield-related QTL were detected in Advanced Backcross-Nested Association Mapping populations of crosses between wild and cultivated barley (Nice et al. 2016). By backcrossing introgression lines with a wild species of rice, F2-populations with improved yield were created (Beerelli et al. 2022). In backcross populations of tomato, the respective wild relative, despite an overall inferior appearance, carried alleles capable of enhancing most traits important to tomato production, including yield-related traits such as fruit size and shape (Tanksley et al. 1996). 

Crop wild relative populations of outcrossing species conserved in their natural habitat have usually undergone many generations of outcrossing. They do not require complex statistical modeling to account for population structure (Capistrano-Gossmann et al. 2017). At the same time, the expected low linkage disequilibrium allows for high resolution mapping (Hansen et al. 2001; Capistrano-Gossmann et al. 2017). Genome-wide association studies scan markers across the genome to find genetic variants associated with phenotypic traits by exploiting pre-existing recombination events within genetically diverse natural populations (Santure & Garant 2018; Arora et al. 2019). While the potential of outcrossing crop wild relative populations for the discovery of monogenic traits relevant for crop improvement was demonstrated (Capistrano-Gossmann et al. 2017), many agronomically important traits are controlled by more than one locus. However, the advantages of these populations have so far not been used for the detection of minor genes underlying quantitative traits.

Sea beets or wild beets [*Beta vulgaris* ssp. *maritima* (L.) Arcang.] are the wild relative of sugar beet. They have a high level of genetic diversity (Renzi et al. 2022; Sandell et al. 2022) and carry many alleles for traits of agronomical importance that were lost during domestication (Panella et al. 2020). The use of wild beets in commercial sugar beet breeding programs is accompanied by several challenges, such as linkage drag, poor agronomic performance, annuality, sterility as well as a wide range of phenotyping challenges. In sugar beet breeding, wild beet populations have been mainly used for the discovery of resistance traits (Capistrano-Gossmann et al. 2017) or discovery of bolting genes so far (Hansen et al. 2001). While few studies evaluated crosses between elite sugar beet material to detect yield QTL (Würschum et al. 2011; Wang et al. 2019), so far, no reports have been made about yield QTL that have been identified and introgressed from wild beet to sugar beet. Most traits that are assessed in yield trials, such as drought tolerance or yield, cannot be properly assessed directly in the crop wild relative (wild beets per se), but require the development of a beet for phenotypic evaluation. This can be achieved by developing mapping populations which contain some amount of elite genome. The question of the optimum amount of elite genome in mapping populations for the detection of quantitative traits within wild beet populations was not yet investigated.

Within this study, we simulated nine different crossing designs to construct mapping populations based on the genotypic data of three different crop wild relative populations. These crossing designs were evaluated for their power to detect phenotype-trait associations for minor genes of complex traits and their false detection rate to explore the optimum amount of elite genome in such mapping populations. Our objective was to determine how QTL detection rate and false positive rate in wild beet populations are affected by (1) the properties of the originating wild beet population (2) the quantitative trait architecture, and (3) the structure of the population used for QTL detection.

## Materials and methods

### Plant material

**Fig. 1:**
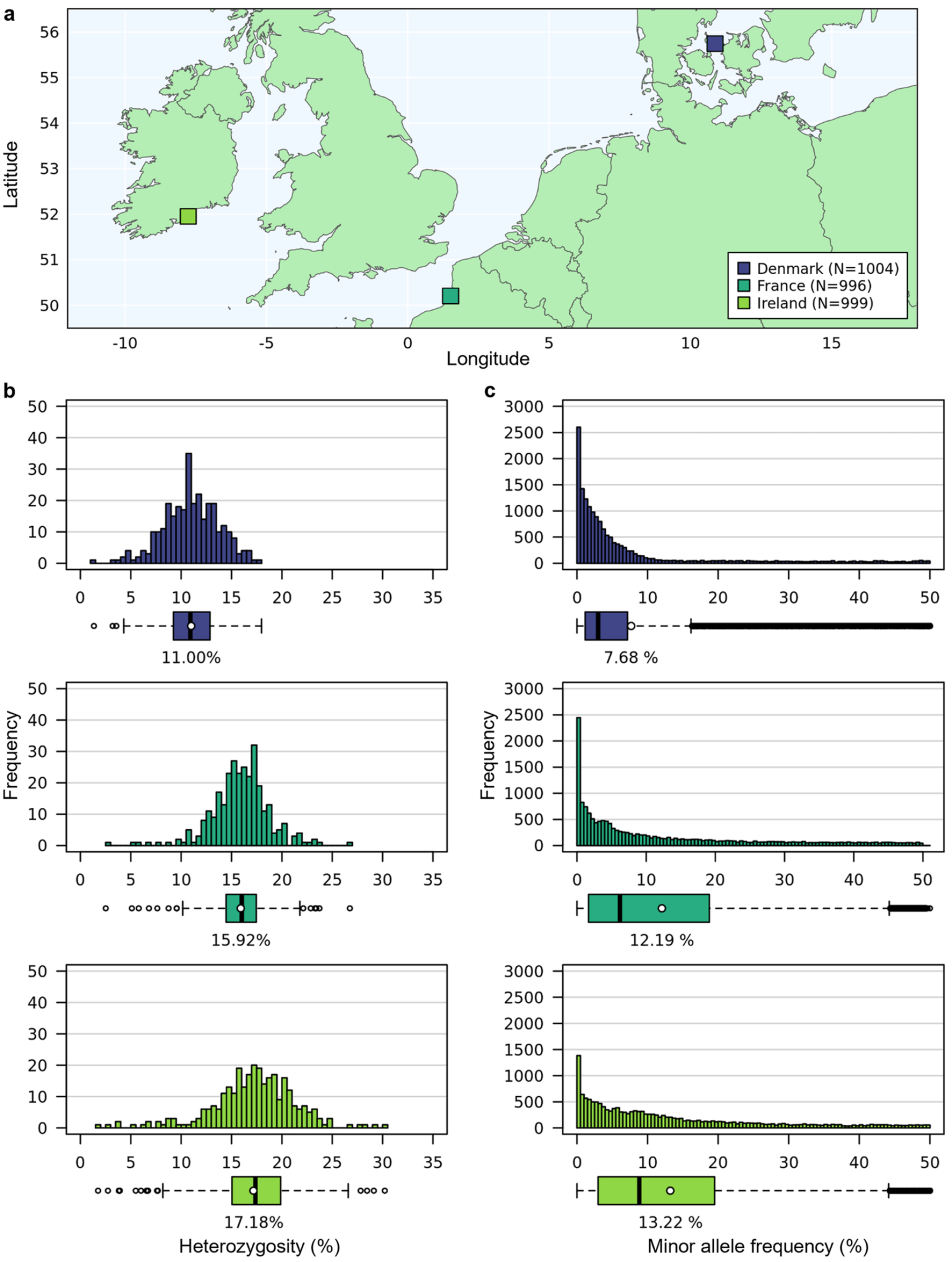
**a** Map showing the locations of the collected crop wild relative populations in Denmark (Kalundborg), France (Brighton), and Ireland (Ardmore). The map was created using the R package “rnaturalearth” (version 1.0.1, Massicotte & South 2023) and “rnaturalearthdata” (version 1.0.0, South et al. 2024). **b** Histograms showing the percentage of heterozygous individuals and **c** the distribution of minor allele frequencies among the selected individuals of all three populations. The colors used for the populations are the same across all plots

We used three wild beet populations sampled across the coastal areas of Europe in their natural habitat in Denmark, France and Ireland (Fig. 1). Populations from these regions have already been described to some extent in other studies (Andersen et al. 2005; Fénart et al. 2008; Capistrano-Gossmann et al. 2017). All populations were chosen due to their location north of the 50th latitude, assuming mainly biannual lifeforms which would facilitate yield trials (Van Dijk et al. 1997).

### Genetic data

Genotyping was carried out for about 1,000 individuals per wild beet population. Additionally genotypic data of one elite line and a tester, both from the sugar beet breeding program of the company KWS SAAT SE & Co. KGaA, were used for the simulation. The elite line was almost fully inbred with ≤ 1% of heterozygous markers and the tester was a CMS sterile within-pool 2-way hybrid with 24.44% heterozygous markers.

All analyses were conducted using R version 3.5.1 (R Core Team 2018). A principal coordinate analysis based on Modified Roger’s Distance was conducted using the R package SelectionTools (v 19.4; https://population-genetics.uni-giessen.de/~software/) which contains the simulation routines of software Plabsoft (Maurer et al. 2008). Genetic outliers were visually identified. A total of nine outliers (Denmark: 3, France: 5, and Ireland: 1) were excluded from the final analysis. Markers with more than two alleles and more than 1% missing values were excluded. Markers that were monomorphic across all three populations and the elite and tester genotypes were also excluded from the analysis. This resulted in the same 16,076 SNPs being used for the simulations across all datasets.

### Phasing

SNP array data generally does not show to which of the two parental chromosomes an allele belongs. For a proper simulation of identical by decent, phasing of the data is necessary to identify alleles co-located on the same gamete. We used BEAGLE (v5.0, Browning & Browning 2007) for phasing. BEAGLE is more accurate than other programs for sample sizes of about 1,000 individuals and high marker density (Browning & Browning 2011). BEAGLE further allows for phasing of data without a phased reference (Browning & Browning 2009). Each wild beet population was phased individually using the remaining individuals of the population as reference. During this process, missing data was imputed by BEAGLE (Browning et al. 2018). The elite and tester were phased separately from the wild beet populations. PLINK (v1.9; Purcell et al. 2007) was used to create the required vcf files.

### Covering the genetic variation of wild beet populations with a subset of size N = 300

A subset of *N* = 300 individuals was chosen from each of the wild beet populations for the simulation. The aim was to cover as much available genetic variation by the chosen individuals. The same set of *N* = 300 individuals was used across all simulation runs.

The individuals were chosen based on haplotypes. For this purpose, haploblocks were build based on a defined window size of five non-overlapping markers using the functions st.def.hblocks and st.recode.hil of the R package SelectionTools.

**Fig. 2:**
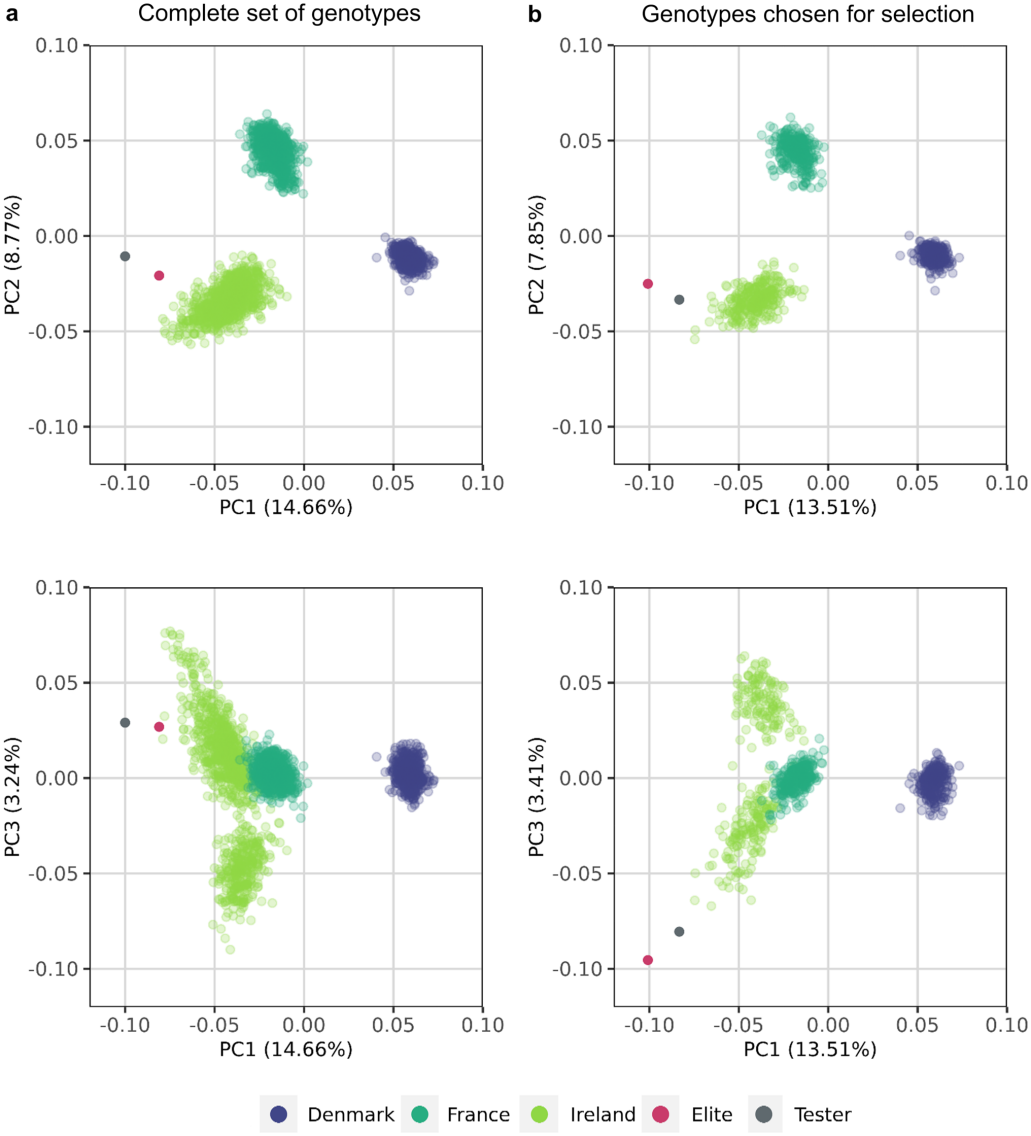
Principal coordinate analysis plot of **a** the complete set of genotypes and **b** the genotypes chosen for simulation. Principal coordinates were calculated as Roger’s distance based on 16,076 SNP loci. The diversity of the genotypes chosen for simulation is representing the diversity present in the complete set of genotypes. The values in parentheses refer to the percentage of variation explained by the principal coordinate

Individuals were chosen in a two-step process: A sub-set of 25 individuals was first chosen based on the rarity of their haplotypes (rarity score = 1 / number occurrences of the haplotype; the sum across all markers was calculated per individual and the individuals with the highest values were selected). In a next step, individuals were added to the set one by one, that contained the largest number of alleles not yet represented in the previously chosen individuals. For this, only haplotypes present at least twice within the population were considered. With this approach, most of the variation for haplotypes present more than once within the populations was covered in the chosen sets of *N* = 300 individuals. The chosen *N* = 300 individuals of each population represent the diversity of the original wild beet populations (Fig. 2).

### Quantitative genetic model

QTL were simulated based on 16,076 SNP markers spread across nine chromosomes for a total length of 625 cM using the package SelectionTools. The average genetic distance between markers was 0.0389 cM with a maximum of 1.838 cM.

We compared three genetic models, simulating either one, two or five QTL with the positive effect assigned to wild beet alleles. A total of 1000 independent simulation runs were performed for each combination of genetic model and wild beet population for all crossing designs. The 1000 simulation runs were conducted to avoid spurious associations with markers. Within each run the QTL were newly assigned.

The total effect sizes of wild beet and elite alleles, as well as their ratio was determined in preceding simulations. These analyses assumed that over time, selection has incorporated numerous small yield-enhancing alleles into elite breeding material (Fulton et al. 1997). Hence, most beneficial alleles originated from the elite genome resulting in its superior performance. As a result, the total elite effects were defined to be twice those of the wild beet effects, which also reflects the average performance observed in field experiments. To differentiate the impact of wild beet and elite alleles, alleles were coded differently for the assignment of effects and during the estimation of genotypic values. A total of three effect files were generated for the analysis.

First, the effect file for the predefined number of QTL (*n* = 1, 2, or 5) originating from the wild beet was created. Previous studies suggest that QTL for complex traits such as yield with larger positive effects exist (Schneider et al. 2002; Reif et al. 2010) and can originate from wild material (Tanksley & Nelson 1996; Reif et al. 2010). However, studies also suggest that most quantitative traits are influenced by a few major-effect QTL, along with additional minor QTL that contribute smaller effects (Tanksley 1993). Hence, a limited number of QTL were simulated, to reflect the expectation that beneficial QTL from wild relative populations are relatively rare (Fulton et al. 1997). For each QTL a random marker was selected from all polymorphic markers within the wild beet population. The allele which the effect was assigned to was determined randomly from both wild alleles present at this locus. The effect of the other wild beet allele was set to 0. All QTL allelic effects were simulated to be additive and of unequal size (Wu et al. 2007), following a geometric series (Lande & Thompson 1990), with each additional QTL having an effect half the size of the previous QTL (eQTL = emax * 1/2n-1). The effect size of the largest simulated QTL (emax) was defined as 5% of the sum of the positive effects of the elite material.

Second, the effect file simulating background noise from the wild beet genome was generated, assuming that a low yield is produced by the wild beets also in the absence of QTL. The total effect size for the remaining markers was set to half the total effects of the elite genome, minus the sum of effects already assigned to QTL.

Third, the effect file for elite alleles was generated. For the elite genome, according to the infinitesimal model (Hayes & Goddard 2001), it was assumed that yield is not based on a few single large QTL but is due to the enrichment with small positive effects over a long period of time. The additive genetic variance was therefore simulated as polygenic variation caused by numerous loci with small effects spread throughout the elite genome. Hence, all markers were assigned equally small effects, with the total elite effects equaling twice the amount of the wild beet effects. The three effect files were combined and used to calculate genotypic values.

### Crossing designs to construct mapping populations

**Fig. 3:**
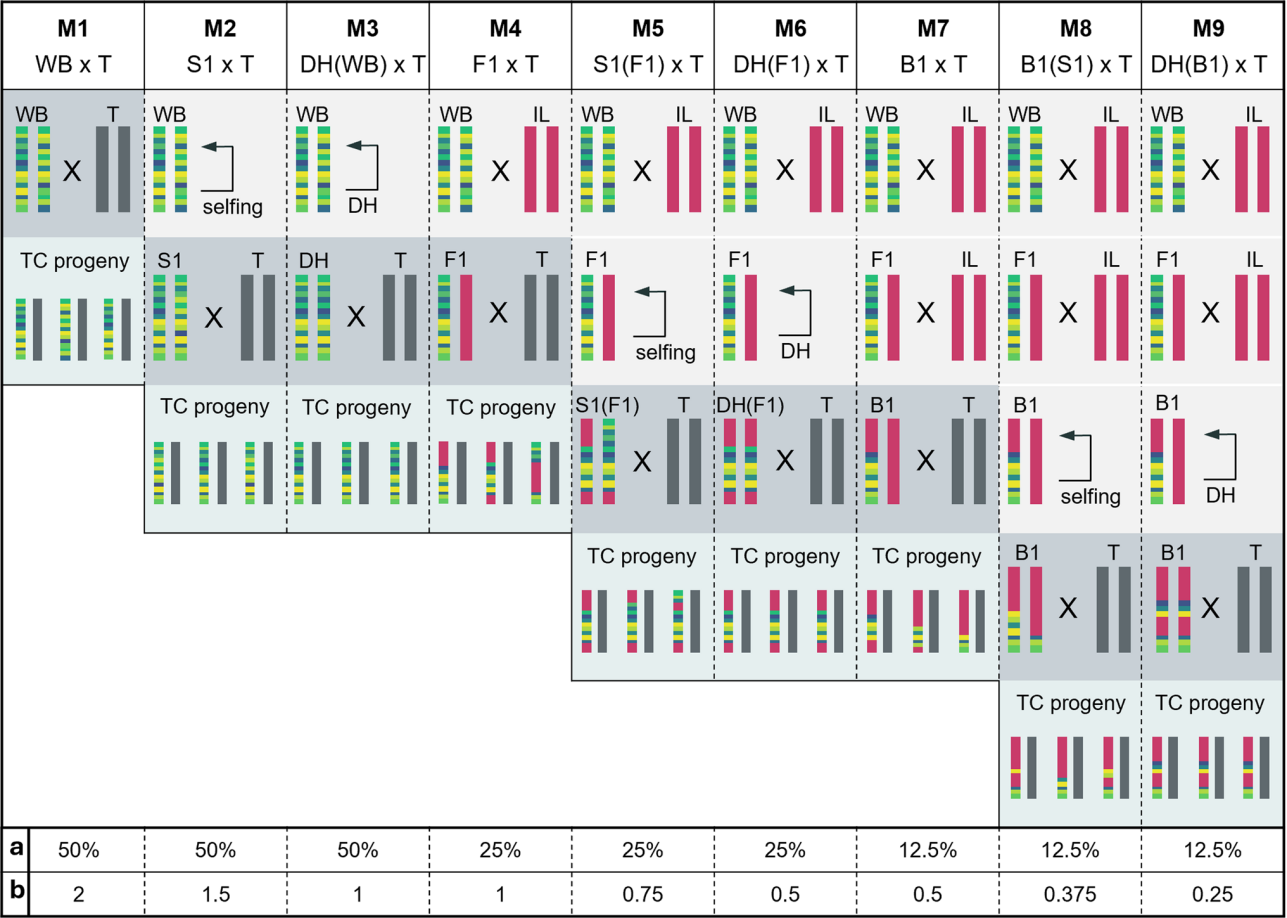
Overview of the nine simulated crossing designs to construct mapping populations (M1 to M9). One exemplary chromosome with a possible segregation of the wild beet genome throughout the cycles of each crossing design is shown. Per wild beet, one random offspring plant per generation is continued. For each entry of the mapping population **a** is giving the average percentage of wild beet genome represented and **b** the average wild beet haplotypes represented. Abbreviations. DH: double haploid, WB: wild beet, T: tester, IL: inbred line, F1S1: selfing of an F1, B1: backcross of an F1 with the elite line, B1(S1): selfing of a backcross

The detection of QTL was evaluated for crossing designs differing in their percentage of elite genome, the amount of wild beet haplotype and the wild beet allele frequency in the mapping population (Fig. 3). Furthermore, the crossing designs differ in the amount of time necessary to develop the mapping populations. For developing the mapping populations, we simulated the following crossing designs M1 to M9 (Fig. 3).

M1: The wild beet genotypes were crossed to a common tester. Every testcross was analyzed as one entry of the mapping population.

M2: The wild beet genotypes were selfed. One random progeny of each selfing was crossed with the common tester.

M3: DH lines were produced from the wild beet genotypes. One random DH line per progeny was chosen and crossed to the common tester.

M4: The wild beet genotypes were crossed to a common elite line. The resulting F1s were crossed with the common tester.

M5: The wild beet genotypes were crossed to a common elite line. The F1s were selfed. One random progeny from each selfing was crossed with the common tester.

M6: The wild beet genotypes were crossed to a common elite line. DH lines were produced for every F1. One DH line per progeny was randomly chosen and crossed to the common tester.

M7: The wild beet genotypes were crossed to a common elite line. The resulting F1s were backcrossed to the same elite to generate a B1. From every progeny, one random B1 was chosen and crossed to the common tester.

M8: The wild beet genotypes were crossed to a common elite line. The resulting F1s were backcrossed to the same elite to generate a B1. From every progeny, one random B1 was chosen and selfed. One random progeny from each selfing was crossed with the common tester.

M9: The wild beet genotypes were crossed to a common elite line. The resulting F1s were backcrossed to the same elite to generate a B1. From every progeny, one random B1 was chosen to produce DH lines. One random DH line per progeny was crossed with the common tester.

All of these crossing designs effectively represent different forms of multi-parent populations with each of the previously selected *N* = 300 individuals from the wild beet population used as parents for one entry of the mapping population. For every test cross progeny, a total of 594 offspring genotypes were simulated.

In total, per wild beet population three different QTL models (*n* = 1, 2 or 5) were simulated for each of the nine crossing designs and 1,000 independent simulation runs were conducted each. The populations were analyzed separately to have independent populations for cross validation of the results.

### Phenotypic values

The genotypic values of the resulting mapping population for each crossing design were determined using SelectionTools. The genotypic value was calculated for each individual of the testcross progeny, by summing up QTL and background effects of the alleles present in the individual based on the previously generated effect files. The mean of the genotypic values of all individuals was used as the mean genotypic value of the entry. The phenotypic values were simulated by adding random nongenetic effects to the known genotypic values. The nongenetic effects followed a normal distribution with a mean of zero and a variance scaled according to the set heritability. The heritability was calculated as broad-sense heritability with h^2^ = v_g_ / (v_g_ + v_e_) with v_e_ being the nongenetic (masking) variance (Bernardo 2004). Based on previous studies on yield QTL in sugar beet (Reif et al. 2010; Würschum et al. 2011) the heritability was set to either 0.5, 0.6, 0.7, 0.8 or 0.9.

### Association mapping

Association mapping was performed for detection of QTL using the R package GenABEL in a two stage process (version 1.8–0; Aulchenko et al. 2007b). In an initial stage, the function polygenic was used to estimate the polygenic model with covariates by maximizing likelihood, providing residuals adjusted for family effects and the inverse of the variance–covariance matrix. For this, the effect of the polygenic term was fitted to the previously simulated phenotypic values.

The mixed model for the initial step of the analysis is given by

$$y_{i} = \mu + \mathop \sum \limits_{j} \beta_{j} c_{ji} + G_{i} + e_{i}$$

where $y_{i}$ is the phenotype of the ith individual, $c_{ji}$ is the value of the jth covariate or fixed effect for the individual i, βj is an estimate of the jth fixed effect or covariate, and $G_{i} { }$ and ${ }e_{{i{ }}}$ are random additive polygenic and residual effects, respectively (Aulchenko et al. 2007a). The random effects are assumed to follow a multivariate normal distribution with mean zero (Aulchenko et al. 2007a).

To adjust for relatedness among individuals a genetic pairwise kinship matrix based on the shared alleles between individuals at all 16.076 SNPs was utilized. Marker to which QTL effects were assigned was excluded from the analysis.

The variance for the polygenic effects is defined as ${\Phi }^{{\sigma_{G}^{2} }}$, where ${\Phi }$ is the relationship matrix and $\sigma_{G}^{2}$ is the additive genetic variance due to polygenes (Aulchenko et al. 2007a). For the residual random effects, the variance is defined as ${\text{I}}^{{\sigma_{e}^{2} }}$, where Ι is the identity matrix and $\sigma_{e}^{2}$ is the residual variance (Aulchenko et al. 2007a). The residuals from this analysis are given by

$$y_{i}^{*} = y - \left( {\hat{\mu } + \mathop \sum \limits_{j} \hat{\beta }_{j} c_{ji} + \hat{G}_{i} } \right) = \hat{e}_{i}$$

where $\hat{\beta }_{j}$ is the estimate of the jth fixed effect and $\hat{G}_{i}$ is the estimated contribution from the polygene (Aulchenko et al. 2007a).

In a second step, a score test for association was performed with the function mmscore, using a linear mixed-effects model approach. The score test is performed using the formula

$$\frac{{\left( {\left( {G - E\left[ G \right]} \right)V^{ - 1} {\text{residual}}Y} \right)^{2} }}{{\left( {G - E\left[ G \right]} \right)V^{ - 1} \left( {G - E\left[ G \right]} \right)}}$$

where G is the vector of genotypes and E[G] is a vector of mean genotypic values, $V^{ - 1}$ the previously estimated inverse covariance-matrix and $residualY$ the residuals from the polygenic model, which were now adjusted for population structure (Chen & Abecasis 2007).

### Evaluation of the simulated crossing designs

The different crossing designs were evaluated for their power to detect phenotype-trait associations for minor genes and their frequency of falsely declaring QTL.

The QTL detection power was evaluated by calculating the true positive rate (TPR) as the number of QTL correctly detected divided by the total number of simulated QTL (in %; TPR = N simulated QTL detected / N simulated QTL). A detection window size around the QTL of ± 2.5 cM was assumed.

The false detection rate (FD) was calculated as percentage of incorrectly detected QTL among all QTL detected (FD = N falsely detected QTL / N detected QTL). If no QTL were detected despite the fact that QTL were simulated, the FD rate was set to 100%. *P*-values were adjusted to control the FD for multiple testing with the fdr method (Benjamini & Hochberg 1995). A threshold of − log10 (p) > 3 was used. An area was identified as a QTL region, if five or more significant markers were located in an area of five cM. Significant markers located next to each other on the chromosome were identified as two separate QTL regions, if there was a stretch of five or more cM without significant markers between these.

## Results

**Fig. 4:**
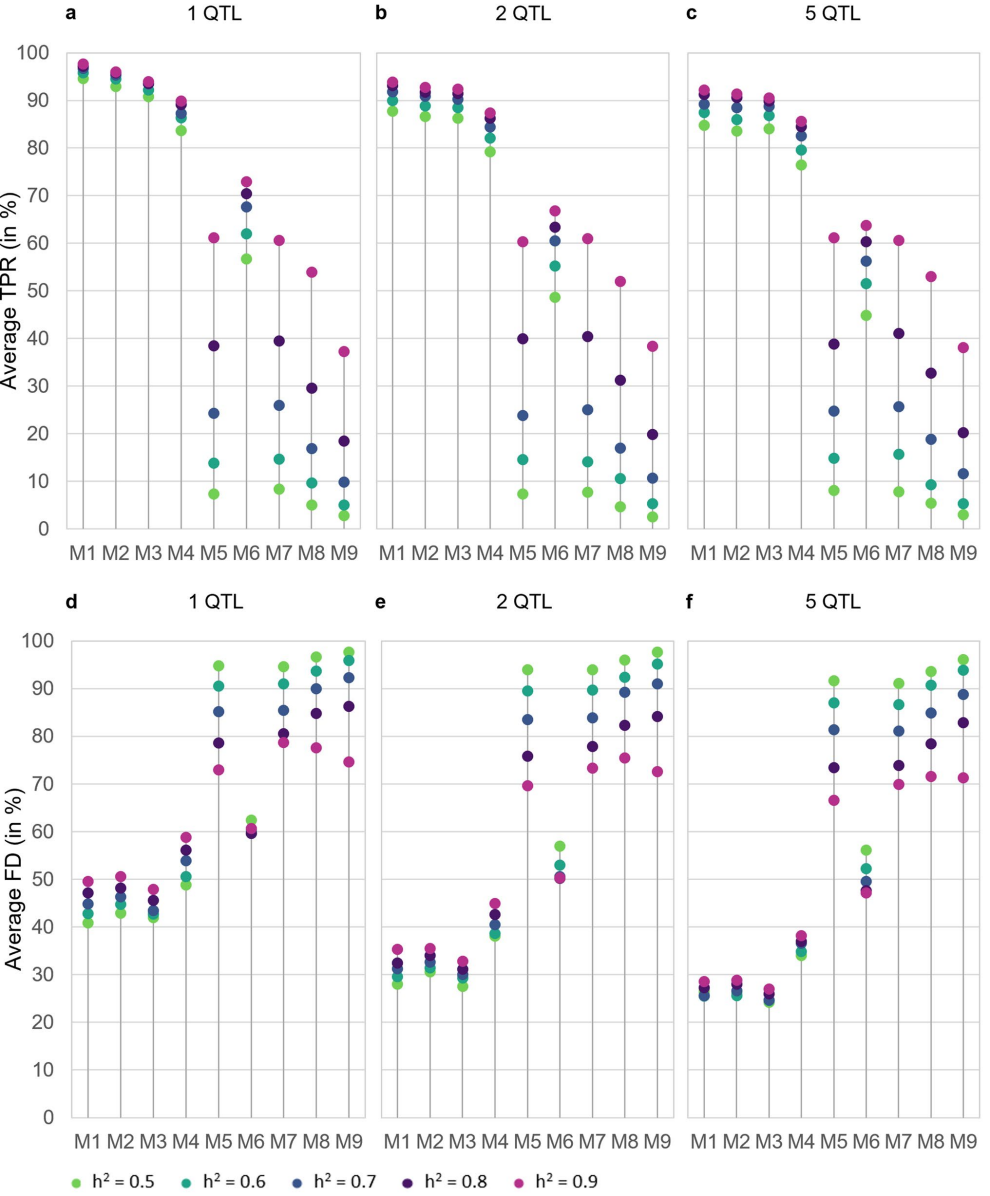
**a**–**c** Average true positive rate (TPR) and **d**–**f** average false detection rate (FD) across all three wild beet populations by crossing design (M1 to M9), number of simulated QTL (1, 2 or 5) and heritability (0.5, 0.6, 0.7, 0.8 or 0.9) in percent

In general, TPR decreases with the percentage of wild beet genome and the amount of wild beet haplotypes represented in the mapping population. TPR is highest for M1 and one simulated QTL (up to 97.60% for h^2^ = 0.9; see. Figure 4a). As we move from M1 to M3, reducing the average wild beet haplotypes represented within the mapping population from two to one (Fig. 3), the TPR decreases slightly (i.e. to 93.97% for M3 and h^2^ = 0.9, Fig. 4a). In M4, despite containing only 25% wild beet genome on average, TPR is still reaching 89.87% for h^2^ = 0.9. As soon as less than one wild beet haplotype is represented in the mapping population (M5 to M9; Fig. 3), TPR drops more severely. While M5 to M7 range between 53.90 and 72.93%, M9 with 12.5% wild beet genome and only 0.25 wild beet haplotypes in the mapping population only reaches a TPR of 37.27% for h^2^ = 0.9.

While TPR also decreases with heritability, crossing designs are affected differently. Reducing heritability only has a small effect on M1 to M3 and TPR is still > 90%, even for h^2^ = 0.5. M4 still has a TPR of 83.70% for h^2^ = 0.5. However, with less than one wild beet haplotype represented within the mapping population (M5 to M9), for h^2^ = 0.5 the TPR is reduced to below 10% (M4, M5 to M9) and only with M6, the TPR is still at 56.7%.

Simulating (several) small QTL next to the 5% QTL also affects QTL discovery of the latter differently across the crossing designs (Fig. 4b, c). For M1 to M4 the TPR is reduced by between 3.50–9.87 percent points and by 9.17–11.83 percent points in M6. When five QTL are simulated in total, there is almost no effect on the TPR of the 5% QTL in M5 as well as M7 to M9.

For M1 to M4 the reduction in TPR for decreasing heritability and simulating multiple smaller background QTL on the TPR of the 5% QTL is of similar size. Heritability affects TPR of the designs M5 and M7 to M9 more severely, while there is almost no effect on TPR when simulating multiple smaller QTL.

FD decreases with the percentage of wild beet genome represented in the mapping population. M1 to M3 with 50% wild beet genome (Fig. 3) show the overall lowest FD while M7 to M9 with 12.5% wild beet genome show the highest FD (Fig. 4d). M4 and M6 are intermediate, while M5 is being an exception and is ranging on a similarly high level as M7 to M9. For M5 and M7 to M9 in many cases no QTL are detected correctly, resulting in very high FD rates of up to 97.63%. In general, the FD decreases with an increasing number of simulated QTL. Especially in M5 and M7 to M9 heritability has a strong effect not only on TPR but also on FD, which increases with decreasing heritability (Fig. 4e, f).

**Fig. 5:**
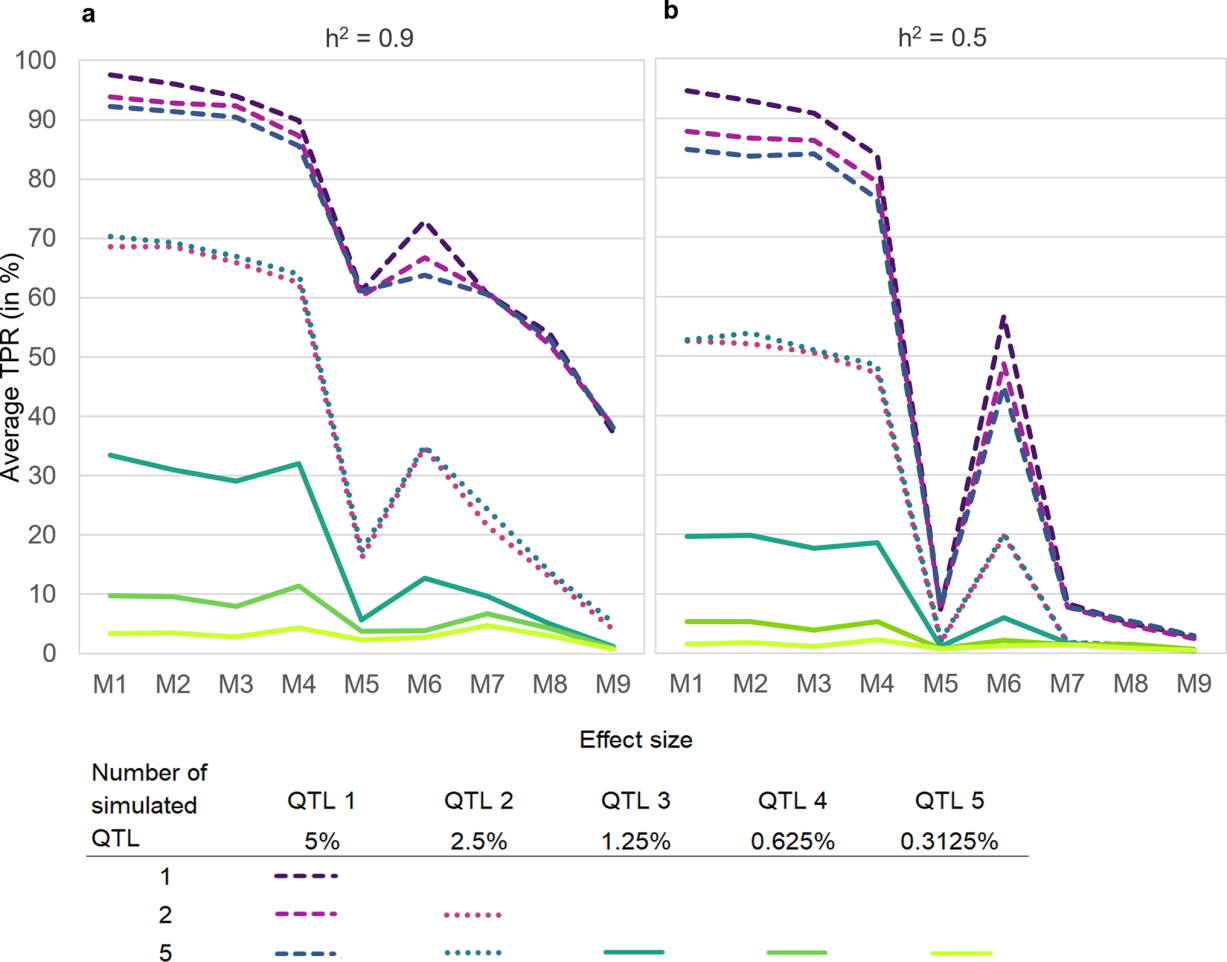
Average true positive rate (TPR) in percent per number of simulated QTL (1, 2 or 5) and corresponding effect size across all three wild beet populations by crossing design (M1 to M9) for a heritability of **a** 0.9 and **b** 0.5

The larger the effect size of the QTL, the higher the TPR (Fig. 5). For a QTL with 5% effect, TPR is overall highest, independent of the number of smaller QTL and heritability in these simulations. For QTL with smaller effect sizes, observations are similar to the observations made for QTL with 5% effect. The TPR is reduced with increasing percentage of wild beet genome and represented wild beet haplotypes within the mapping population from M1 to M9. TPR of M5 is breaking down compared to M4 and M6 for all effect sizes. TPR is also reduced with heritability. QTL with 2.5% can still be found with the crossing designs M1 to M4 with a probability of 62.53–70.30% when heritability is at 90%. However, when heritability is reduced, the TPR is reduced to less than 52% for these crossing designs. With all other crossing designs, TPR of a 2.5% QTL reaches 35.03% at best for M6 with h^2^ = 0.9. TPR is reduced to almost 0% for other crossing designs and lower heritability. The effect of simulating additional smaller QTL on the TPR of the 2.5% QTL is very low. TPR of the 2.5% QTL when simulating two QTL or five QTL are very similar.

QTL with smaller effect sizes will most likely not be detected, independent of heritability and crossing design of the mapping population. All QTL with an effect size of ≤ 1.25% have a TPR of 33.47% at best (M1 at h^2^ = 0.9) and close to 0% for smaller effect sizes. With a lower heritability (i.e., h^2^ = 0.5), TPR is reduced to below 20% and goes down to almost 0 in many cases.

**Fig. 6:**
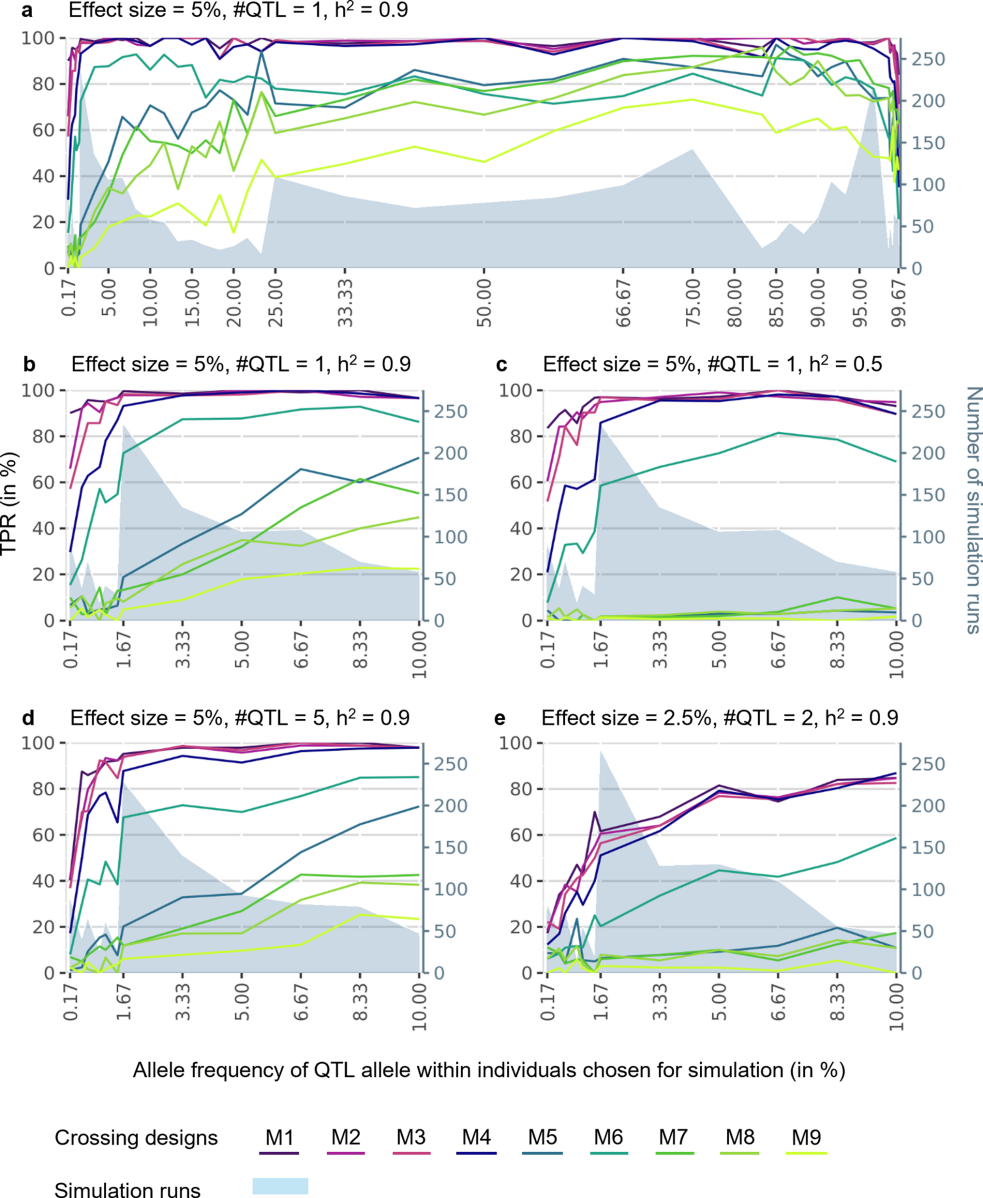
Number of simulation runs and corresponding true positive rates (TPR) of different classes of allele frequencies of the QTL allele across all simulation runs and wild beet populations for different heritabilities (h^2^), effect sizes and number of QTL simulated (#QTL). Allele frequencies shown are those of the individuals chosen for simulation

Depending on the simulated crossing design, QTL can be discovered, even if the QTL allele has a low frequency within the wild beet individuals chosen for simulation (Fig. 6). In general, the TPR is higher if the frequencies of both wild beet alleles are balanced. If the allele carrying the QTL effect is present with a low frequency within the wild beet individuals, TPR decreases. At which allele frequency the TPR starts to decrease and how quickly it drops down how far, depends on the simulated crossing design. In M1, with 50% of wild beet genome and both wild beet haplotypes represented in the mapping population, the TPR only decreases slightly. Even in the most extreme cases simulated within this study with an allele frequency of the QTL allele of 0.17% (equal to only one allele present in all 300 individuals), the TPR is still by 90.11% on average (Fig. 6a, b). The required frequency of the QTL allele within the chosen wild beet individuals to reach a TPR of ≥ 90% increases with an increasing percentage of elite genome in the mapping population. In M4 with one wild beet haplotype and around 25% wild beet genome represented (Fig. 3) an allele frequency of 1.67% (M2: 0.50%, M3: 1.00%) or higher is required to achieve a TPR of about 90%. In M9 the TPR does not exceed 73.24% in any case, even with high frequencies of the QTL allele. When the QTL allele has a frequency of ≥ 5% within the wild beet individuals chosen for simulation, TPR of crossing designs M1 to M4 are similar (~ 100%).

If heritability is reduced, in all crossing designs a higher frequency of the QTL allele is required to reach a certain TPR (Fig. 6c). A TPR of 90% requires at least an allele frequency of 0.67% in M1, 1.00% in M2, and 1.17% in M3. With an allele frequency of the QTL allele of 3.33%, a TPR of 95% can be achieved with M4. TPR does not exceed 10% for M5 or M7 to M9 for allele frequencies of the QTL of 10% or below.

Increasing the number of simulated QTL increases the QTL allele frequency required for QTL discovery (Fig. 6d). Even with a heritability of 0.9, in M1 an allele frequency of 1.17% is required to reach a TPR of 90%. With an allele frequency of 0.17%, only a TPR of 40.23% is reached with M1.

The described trends hold true for an effect size of 5%. If QTL are smaller, the general trend is similar, however on an overall lower level. Also, maximum TPR reached are lower. With smaller effect sizes, the required QTL allele frequencies increase severely (Fig. 6e). If two QTL are simulated with a heritability of 0.9, even with a frequency of the QTL allele of 10%, only a TPR of 84.78% is reached with M1.

In general, decreasing heritability, increasing number of simulated QTL, increasing amount of introduced elite genome and a decreasing effect size of the QTL allele are all factors that cause for an increasing allele frequency of QTL allele required to reach desired TPR. However, an increase in number of simulated QTL appears to affect TPR less than decreasing heritability or increasing the elite genome. All trends described above are observed across all three populations independently.

## Discussion

The main objective of this paper was to compare different crossing designs concerning their power to detect minor QTL of quantitative traits in wild beet populations, which cannot be evaluated in the wild beet per se*,* such as yield or drought stress.

### Population structure

For wild beet populations increasing homogeneity within the material is not found to be beneficial in terms of increasing power (Fig. 4). *Beta vulgaris* ssp. *maritima* (L.) Arcang. is a naturally outcrossing species with a high degree of self-incompatibility and hence usually high levels of heterozygosity within the populations (Hautekeete et al. 2020; Felkel et al. 2023). Contrary to that, the individuals of all the populations we used are homozygous at 69.78–98.66% of all loci under evaluation. Between on average 11.00% of the markers in the population from Denmark and 17.18% in France are heterozygous (Fig. 1). Selfing reduces the heterozygosity by half. In this case this would correspond to an increase from 11–17% to only ~ 5–9% on average in homozygosity. Double haploid production leads to a homozygous state within one generation. However, increase in homozygote loci by only 5–17 percent points at most appears to not improve homogeneity of the mapping population and therefore phenotyping accuracy enough to have a major impact on QTL detection power. The increase in homozygosity via selfing or DH production adds additional steps to material development, which increases development time, but does not increase TPR. This is independent of the percentage of elite genome within the mapping population.

With a decrease in the percentage of wild beet genome and wild beet haplotypes present in the mapping population, there is a trend for TPR to decrease. M6 is an exception to this trend. M1 to M3, which contain 50% wild beet genome in the mapping population, have the highest TPR. All crossing designs in which elite genome was introduced, except for M4, have severely lower TPR. As soon as less than one full wild beet haplotype is represented within the mapping population (M5 to M9), TPR drops severely to below 20% for h^2^ = 0.9 and down to 0% with h^2^ = 0.5 (Fig. 4).

One reason for this may be that the probability to lose wild beet alleles during material development increases as the proportion of the wild beet genome in the mapping population decreases. This also increases the risk of reducing the frequency of the QTL alleles or losing them completely and hence lowers the TPR. Wild beet genome may be masked by increasing the percentage of elite genome, since in association mapping alleles that are identical by descent cannot be differentiated. SNP array data generally does not show to which of the two parental chromosomes an allele belongs. Hence, while the SNP allele coming from the wild beet may cause the effect, the same SNP allele coming from the elite genome may not, which may cause problems with the associations. With M4 to M9, by crossing wild beet to elite, genomes are “mixed” and cannot necessarily be differentiated in genotyping. Clear associations of alleles to either wild beet or elite parent could improve power.

Further, there may also be variation between entries caused by the elite genome represented in the entries of the mapping populations. Whereas in M4, there is only one wild beet and one elite haplotype present in the mapping population, this does not hold true for crossing designs M5 to M9. In these crossing designs, different percentage and parts of the wild beet and elite haplotypes may be represented within the genotypes of each entry of the mapping population. This may cause for additional variation that further reduces the precision of the phenotyping and hence power. This does not hold true for the crossing designs involving DH production. In these crossing designs, the only variation remaining in the mapping population is caused by the tester genome. On the other hand, the crossing designs involving selfing steps have unbalanced allele frequencies within the mapping populations. While some of the genotypes within one entry are fixed for the wild allele, some are heterozygous, and others are fixed for the elite allele. Hence, some parts of the wild beet genome will not be represented at all in some of the genotypes. This segregation causes for a higher phenotypic variation and results in a lower power to detect QTL, especially in M5. In M8, the drop in TPR is not as extreme compared to the corresponding designs M7 and M9. This may be due to the fact, that there is a higher percentage of elite genome present in M8. The elite line contains less than 1% heterozygous markers, so a higher percentage of elite genome causes for a higher homogeneity compared to M5.

Heritability in this paper is simulated in the range of 0.5–0.9. This is similar to the range of heritability for yield related traits in sugar beet observed in field trials (Schneider et al. 2002; Reif et al. 2010). With decrease in heritability, a decrease in power to detect QTL can be observed (Figs. 4, 5). This trend has also been observed in other studies (Reif et al. 2010). Heritability affects the power of crossing designs M5 and M7 to M9 quite strongly, whereas crossing designs M1 to M4 and M6 are a lot less affected. This may also be due to the higher phenotypic variation caused by the elite genome in M5 and M7 to M9.

Despite the fact, that in M4 the percentage of wild beet genome is reduced by 50% compared to M1 to M3, the TPR is still on a similar level as for those designs, especially, when multiple QTL are simulated (Fig. 4). While the TPR in M6 is overall lower, it is still high compared to M5 and M7 to M9, especially for lower heritabilities. In M4 and M6 the variation caused by differing haplotypes and percentage of the elite genome represented within different entries is limited compared to M5 and M7 to M9. Further, in M6, due to DH production, the entries of the mapping population are homogenous. The only remaining variation within the entries of the mapping population is caused by the tester genome. Overall, this may increase phenotyping accuracy and hence power in M4 and especially in M6. Therefore, this may be an explanation for the limited influence of heritability on TPR for these crossing designs. However, the additional step of DH production in M6 entails a higher risk for loss of the QTL allele during material development. This seems to outweigh the advantages of homogeneous entries, less variation and increased phenotyping accuracy in the mapping population in M6 and may explain the reduced TPR compared to M4.

Overall, the TRP in wild beet populations is influenced by several factors. Crossing designs M1 to M4 with a higher percentage of wild beet haplotypes seem to be preferable and have the highest TPR. Additional steps to increase homogeneity within the mapping population in M2 or M3 have no beneficial effect on TPR compared to M1.

In addition to the low TPR, M5 to M9 also show high FD rates. The high FD rates of these crossing designs result mainly from not identifying any QTL at all, despite QTL were simulated, less from falsely identifying QTL. M1 to M4 are hence also favorable in terms of low FD rates compared to M5 to M9.

### Trait architecture

No negative QTL were simulated for the wild beet genome, but an overall inferior performance. Falke and Frisch (2011) showed in simulations with a donor carrying positive as well as negative alleles for the trait under consideration, that unless the loci carrying the alleles are closely linked, the presence of negative alleles does not hinder the detection of positive alleles for the trait under consideration. Since linkage disequilibrium in wild beet populations is very low (Capistrano-Gossmann et al. 2017), this was not regarded in these simulations.

QTL explaining 5% of the sum of the genotypic effects can be detected with at least some of the crossing designs such as M1 to M4 and with a lower probability also with M6. QTL with an effect of about 2.5% can only be detected with M1 to M4 with a probability of 60–70% and a high heritability. With a low heritability or any other crossing design, QTL with these effect sizes are most likely not detected under the simulated conditions. QTL with effects smaller than 2.5% are most likely not detected, regardless of the simulated type of material development and heritability as the results of this study show (Fig. 5). This also corresponds to other studies, which found that for smaller effect sizes, the required heritability (Falke & Frisch 2011) or sample size to reach sufficient power increases (Spencer et al. 2009; Visscher et al. 2017).

QTL with a small effect of ≤ 2.5% will most likely not be pursued further or used within practical breeding programs per se. Nevertheless, it is important to evaluate the influence of their presence on the detection of larger QTL. The presence of smaller QTL has an influence on the power to detect QTL in some of the simulated crossing designs. For M1 to M4 and M6, a reduction of up to ten percent points in TPR can be observed, if smaller QTL are simulated additionally to the 5% effect QTL (Fig. 5).

Within this study, QTL are assigned at random and therefore more than one QTL may by chance be assigned to the same chromosome or even to a similar position. If multiple QTL are assigned to a similar region on a chromosome, the variance explained by this interval is increased and thus the power to detect a QTL in this interval (Stich et al. 2005). Hence, the presence of a smaller QTL may increase the chance of finding a larger QTL. However, while the chance to identify this region may be increased, it may not be possible to determine the exact number of contributing QTL (Stich et al. 2005). Consequently, the number of QTL associated with a trait influences the power of QTL detection and the overall power decreases with an increasing number of QTL. These effects appear to be more pronounced when heritability decreases. This has also been observed and described in other studies (Bernardo 2004).

### Characteristics of wild beet populations

The wild beet populations used in this study all exibit a low heterozygosity (on average 11.00–17.18%), low minor allele frequencies (between 7.68% and 13.22% on average, Fig. 1) and due to many generations of outcrossing a low linkage disequilibrium.

Due to the already low heterozygosity observed within these populations, increasing homozygosity within the material further is not found to be beneficial in terms of increasing power. Rather, this increases the risk of losing the QTL alleles. Considering the low average minor allele frequencies within these populations, most alleles are not represented in many individuals. The less frequent alleles are, the more likely they are lost during material development (Reif et al. 2010; Lou et al. 2020). Only if the QTL allele is still present in the mapping population, the QTL can be detected. With crossing designs M1 to M4, QTL can be discovered, even if the QTL allele has a frequency of 0.17% (equal to 1 allele in 300 genotypes) within the wild beet individuals chosen for simulation. The risk to lose alleles is higher, the less wild beet genome and the less wild beet haplotypes are represented within the mapping population, especially when no further steps for optimization are carried out.

The power to identify a true association between a SNP and trait with association mapping depends on the phenotypic variance within the population explained by the SNP (Korte & Farlow 2013). The phenotypic variance is determined by how strongly the two allelic variants differ in their phenotypic effect size, and their frequency. Therefore, rare variants and small effect size both present problems for association mapping. Rare alleles are present in only few individuals, which reduces power. When the QTL allele is rare, the power to detect the QTL is low, unless the effect and sample sizes are large (Spencer et al. 2009; Wang & Xu 2019). The smaller the effect, the larger the required sample size to reach sufficient power, especially when the QTL allele is rare within the population (Zondervan & Cardon 2004; Spencer et al. 2009; Visscher et al. 2017).

The use of genetic resources such as wild relative populations with a low extent of linkage disequilibrium between markers (Capistrano-Gossmann et al. 2017) can increase mapping resolution, also in chromosomal regions with an otherwise high degree of linkage disequilibrium (Hansen et al. 2001). By taking advantage of the recombination events accumulated over a long period of time within the wild beet population, the number of samples necessary to achieve a given resolution is greatly reduced (Hansen et al. 2001). This may explain why alleles present only in very few individuals of the population can still be found in the crossing designs that cover a large percentage of the wild beet haplotypes in the resulting mapping populations.

### Practical aspects of breeding with crop wild relatives

Crossing designs containing a large percentage of wild beet genome may face some practical issues, that are not considered within the simulations of this paper. Traits from wild beets, such as annuality or a tendency towards bolting, forked beets and so forth, can mask yield potential by causing problems within field trials and hence with measurement of yield related traits. Especially when considering lethal alleles carried within the wild beets, crossing designs where wild alleles are present only in a heterozygous state in the mapping population (M4 and M7) may be beneficial.

Practical experience shows that at least 75% elite genome in the mapping population is needed, to allow for reasonable phenotyping within yield trials. Material containing more than 25% wild beet genome does not produce proper beets, but often forked beets or has a tendency to bolting. Bolting plants use most of their energy to produce seed rather than the desired beet. While the extent of this depends also on the frequency of annuality within the wild beet population, populations from Northern Europe, as are the ones regarded within this study, in general have a lower tendency for bolting. They are located north of the 50th latitude and hence annual bolting behavior is assumed to not be the dominating life form (Van Dijk et al. 1997). Populations from more Southern regions on the other hand can contain extremely high frequencies of annual genotypes. On the other hand, forked beets are extremely difficult to harvest mechanically. While a manual harvest is theoretically possible, this will largely increase trial cost. Both, forked beets and bolting, hence impact yield trials and make phenotyping for yield in practice very difficult.

Overall, a minimum of 75% elite genome is therefore recommended to enable proper yield trial with this type of material. This is also found in studies with other crops such as wheat, where lodging can mask yield potential (Schulthess et al. 2022). Producing F1s with elite lines reduces this risk and allows for phenotyping in the field (Schulthess et al. 2022). Therefore, despite the fact that M1 to M3 have the overall highest TPR, they cannot be recommended for practical application in traits which require proper beet development for phenotyping and when frequency for bolting and forked beets within the material is high.

Further issues from working with wild beets may arise and impact the feasibility of some of the simulated crossing designs. Wild beets do not generally allow for selfing due to various reasons, one of them being self-incompatibility (Hautekeete et al. 2020). This can lead to strong problems with seed production in crossing designs, that involve selfing steps, especially, if no elite genome is introduced as in M2. Also, low pollen production, sterility, problems with bolting or with the flowering time adjustment can pose strong difficulties on material development. These issues may impact seed production and seed availability and may be more pronounced in designs with a small percentage of elite genome. Within this study, for every entry of the mapping population a total of 594 genotypes were simulated, representing six plots with 99 beets per plot. This represents a normal plot size in practical breeding and is similar to what has been done in other studies for yield related traits in sugar beet (Schneider et al. 2002; Würschum et al. 2011). More plots would most likely improve the accuracy of estimation of yield parameters. However, even the amount of seed required for six plots may be difficult to obtain based on only one plant as pollen parent, especially from wild plants without any elite background. With those crossing designs in which the percentage of elite genome is higher, these problems will be reduced, nevertheless the available seed will be limited by being based on one plant as a pollen parent. Especially within the crossing designs involving DH production the number of progeny could be larger, but this would also require more input. Production of DH lines based on wild material may also be difficult.

In summary, various practical factors influence different approaches to material development. Since these aspects are not accounted for in the simulations, they are not directly reflected in the results but should be taken into account for interpreting the findings. Although it requires one additional material development cycle compared to M1, M4 offers the best balance between maintaining wild allele representation and ensuring proper phenotyping, as it includes a sufficient percentage of elite genome to reduce undesirable traits while still preserving rare wild alleles for QTL detection. Further, the potential challenges of selfing during seed production strongly support selecting a crossing design like M4.

### Methodological aspects

The power to detect QTL is also influenced by sample size (Spencer et al. 2009; Wang & Xu 2019)**.** The simulations in this study are based on 300 genotypes per wild beet population. Developing a mapping population based on all 1,000 genotypes would be very costly and labor intensive. Since not all genotypes provide new genetic variation due to relatedness, a subset of N = 300 genotypes was chosen from each of the wild beet populations for the simulation. This number is based on population sizes used for association mapping in other studies in sugar beet (Reif et al. 2010) or studies based on wild beet populations (Capistrano-Gossmann 2017). The genotypes were chosen based on haplotypes rather than on allelic diversity, since the choice of SNPs might be biassed and inhibit capturing all the variation existing in the exotic material. Using a defined number of markers rather than haploblocks based on linkage disequilibrium allows for applying the same procedure across populations and better comparison of results. Haplotypes that were present only once within the entire population have a high possibility of being errors, such as genotyping errors, imputation errors or phasing errors. These haplotypes were not discarded because they might also constitute rare alleles. However, they were not specifically considered in the process of choosing genotypes for simulation, which was restricted to haplotypes present at least twice in the population.

Effects caused by alleles contributed by the tester are not considered in the genotyping step, since genotyping in this simulation is carried out prior to crossing with the tester. However, the alleles from the tester also affect the phenotype. This discrepancy causes variation which is unaccounted for and hence adds to the error variance and reduces power. However, this issue applies to all simulated crossing designs similarly.

In this study, the QTL allele is removed from the analysis to simulate that the loci carrying the QTL allele is usually not covered by a SNP and hence not genotyped. However, it may be linked to another marker that is indeed genotyped (Zondervan & Cardon 2004). Indirect association studies make use of the principle, that markers which are close to a QTL allele on the same chromosome will be more often co-inherited than expected under independent assortment (Zondervan & Cardon 2004). Therefore, the power of detection is influenced by the linkage disequilibrium between the genotyped markers and the locus carrying the QTL allele as well as the marker density. Especially with a low linkage disequilibrium, the marker density needs to be sufficiently high to achieve reasonable power to detect association between genotyped markers and the QTL allele (Santure & Garant 2018).

In this study, an area was identified as QTL if five or more significant markers were located in a stretch of five cM. Two such regions were identified as different QTL, when a stretch of five or more cM without significant markers separated these. This approach is based on practical experience and represents a minimum requirement of what would be followed up in practical breeding programs.

QTL discovery can be influenced by the marker density of the region of the chromosome in which the QTL allele is assigned. In this study, the average genetic distance between markers was below 0.05 cM on all chromosomes. Hence, the impact of this is assumed to be limited.

In this paper a few assumptions were made for simplicity, which not necessarily always hold true in practical application. For one, a purely additive model was used. Dominance or epistatic effects were not regarded.

A significant effect of dominance variance in the inheritance of root yield and sugar content with a less significant effect of the additive effects was observed in some studies (Stancic et al. 2014). However, other studies found additive genetic variances to be considerably larger than epistatic and dominance variances for root yield in sugar (Kristensen et al. 2023). If parental lines are not fully inbred or when regarding three-way hybrids, additive genetic variance explains the largest portion of the variance (Kristensen et al. 2023).

The power to detect epistasis with testcross performance is low due to masking effects from the tester (Gallais & Rives 1993). Further, Reif et al. (2010) argue that in cases where the genetic contribution of the genotypes to the testcross progenies is 25%, the relative contribution of additive variance to the genetic variance among testcross progenies is eightfold larger than the additive-by-additive variance. This results in a lower power to detect epistatic effects compared to main effects. These results are in accordance with the results reported for testcross performance of complex traits in other cross-pollinating species such as maize (Mihaljevic et al. 2005). Reif et al. (2010) therefore conclude, that epistasis may be ignored in sugar beet breeding. In this study, in crossing design M1 to M3, the contribution of the wild beet genome to the test cross progeny is 50%. For all other crossing designs the contribution of the wild beet genome within the test cross progeny is 25% or below. Therefore, epistatic effects were not considered in this study either.

Within this study, the costs of different approaches for material development were not regarded. These do not only differ strongly for different organizations, but also may change rapidly over time with new technology evolving. That said, crossing designs including DH production, such as M3, M6, and M9, are likely to lead to higher material development cost, assuming that DH development is possible. The different crossing designs also vary in the time required to create a mapping population. It can also be assumed that crossing designs which require multiple steps of material development also produce higher cost than crossing designs based on fewer development steps.

## Conclusion

QTL originating from the wild beet genome can be found with some of the simulated crossing designs, while others are not suitable for QTL detection under the simulated conditions within wild beet populations. Due to the low minor allele frequency, the largest risk to detection power is the loss of QTL alleles during material development. Under the assumptions within this study, QTL detection is most effective when assessing wild beet directly in testcrosses, making crossing design M1 preferable for minimal resource investment.

However, field trials require at least 75% elite genome to mitigate unfavorable wild traits like bolting and forked beets, which could obscure trait evaluation. Increasing elite genome improves phenotyping but lowers wild allele frequencies, reducing association mapping power. The number of QTL affects detection power, with slight reductions when multiple QTL with small effects are present. M1 is best for detecting rare alleles (< 1%), while M4 can still identify those with ~ 1.5% frequency, even under lower heritability. Overall, M4 offers the best balance for detecting yield-related QTL, providing sufficient elite genome for proper beet development while maintaining representation of wild beet haplotypes to preserve low-frequency alleles for QTL detection.

These findings offer valuable insights into optimizing QTL detection and crossing designs based on wild beet populations. This also highlights the need to protect and conserve these populations in their natural habitats to ensure their availability for future breeding efforts. A similar approach could be applied to other predominantly outcrossing species such as rye or sunflower, where allogamous flowering promotes the accumulation of recombination events over successive generations within crop wild relative populations. Further research is required to refine these strategies for different species and assess their feasibility in practical breeding programs.

## Data Availability

The genetic data and code are available from the authors upon reasonable request.
